# Soil Contamination with *Toxocara* Eggs in Public Schools in Rural Areas of Southern Thailand

**DOI:** 10.1155/2020/9659640

**Published:** 2020-09-08

**Authors:** Nonthapan Phasuk, Ratee Kache, Kanjana Thongtup, Saowalee Boonmuang, Chuchard Punsawad

**Affiliations:** School of Medicine, Walailak University, Nakhon Si Thammarat, Thailand

## Abstract

Soil is considered the primary source of *Toxocara* transmission to humans, especially children. The status of soil contamination with *Toxocara* eggs in southern Thailand is unknown. This study aimed at estimating the soil contamination with *Toxocara* eggs in public schools in Nakhon Si Thammarat province in southern Thailand. Soil samples were collected from 12 public schools between August and September 2017. At each site, ten soil samples were collected from the following five types of locations: (1) playgrounds, (2) football fields, (3) sidewalks, (4) schoolyards, and (5) areas around cafeterias. In total, 120 samples were examined for *Toxocara* eggs with a modified flotation method using a sucrose solution. *Toxocara* eggs were detected in 8 (66.7%) of the 12 studied public schools. Of the 120 soil samples, 22 (18.3%; 95% CI: 11.9, 26.4) were contaminated with *Toxocara* eggs. The highest levels of *Toxocara* egg contamination were observed in playgrounds (41.7%; 95% CI: 22.1, 63.4), followed by football fields (20.8%; 95% CI: 7.1, 42.2), sidewalks (12.5%; 95% CI: 2.7, 32.4), and schoolyards (12.5%; 95% CI: 2.7, 32.4). There were significant differences in the distribution of *Toxocara* eggs across location types (*p* < 0.05). The findings demonstrated that the soil samples from public schools were contaminated with *Toxocara* eggs. Playgrounds were the most heavily contaminated locations. Teaching children proper handwashing steps and discouraging geophagia should be implemented to reduce the distribution of *Toxocara* and limit future *Toxocara* infections.

## 1. Introduction

Toxocariasis is a neglected parasitic infection caused by the larval stage of *Toxocara canis* (dog roundworm) and *Toxocara cati* (cat roundworm), which are intestinal parasites of dogs and cats, respectively [1]. Humans can be infected by accidentally ingesting embryonated *Toxocara* eggs shed by definitive hosts and through the consumption of third-stage (L3) larvae in raw or undercooked paratenic hosts [2, 3]. Children have the highest risk of toxocariasis due to their behavior, such as eating soil, putting objects in their mouth, and eating earthworms; their close contact with infected dogs; and their poor hygiene habits [4, 5]. The clinical manifestation of *Toxocara* infections in dogs is associated with the stage of infection. During larval migration through the lungs, dogs may develop a cough, nasal discharge, pneumonia, and pulmonary edema, while during adult worm infection, it may cause mucoid enteritis, vomiting, diarrhea, ascites, anorexia, and anemia [6]. *Toxocara* infections in humans can cause four primary clinical syndromes according to the organs affected: visceral larva migrans (VLM), a systemic disease caused by larval migration through major organs; ocular larva migrans (OLM), a disease limited to the eye and optic nerve; neurotoxocariasis (NT), a syndrome related to larval migration in the central nervous system that causes meningitis, encephalitis, cerebral vasculitis, and/or myelitis; and covert/common toxocariasis, a nonspecific syndrome with nonspecific symptoms, wheezing, and eosinophilia [5, 7–10]. However, the vast majority of human *Toxocara* infections are asymptomatic, and serological testing using an enzyme-linked immunosorbent assay has sufficient specificity to be the best indirect test for diagnosing this infection [7, 11]. A previous systematic review revealed that the estimated global *Toxocara* seroprevalence in humans was 19% [12]. However, our recent study revealed that the seroprevalence of *Toxocara canis* infection in primary school children in rural southern Thailand was as high as 58% [13].

The transmission of the *Toxocara* parasite occurs when an infected dog or cat sheds eggs in their feces in public parks or playgrounds. It takes 2–4 weeks for the larvae to develop and for the eggs to become infectious [4]. Consequently, soil contamination seems to be the most direct indicator of risk to human populations. A systematic review showed that the pooled global percentage of *Toxocara* egg contamination in public places was 21% [14]. Many previous studies carried out in different countries demonstrated that the contamination rate of *Toxocara* eggs ranged from 5 to 70% in soil in public parks, playgrounds, sandpits, beaches, backyards, sidewalks, and roadsides [8, 15–26]. Embryonated *Toxocara* eggs have been recovered from the hair of dogs [27, 28], which demonstrates that direct human-dog contact could also be a route of infection for humans. In southern Thailand, a previous study by Uga and colleagues revealed a 19% soil contamination rate with *Toxocara* eggs in Songkhla province [29]. However, the current status of soil contamination with *Toxocara* eggs in Thailand is still unclear and should be investigated further.

Currently, there are large numbers of stray dogs and cats in urban and rural areas of southern Thailand; these animals can easily access public areas. Furthermore, keeping animals as pets has increased in popularity. These factors may contribute to soil contamination with *Toxocara* eggs. Therefore, this study was designed to estimate the extent of soil contamination with *Toxocara* eggs in public schools in Nakhon Si Thammarat province in southern Thailand.

## 2. Materials and Methods

### 2.1. Study Area

Nopphitam is a district of Nakhon Si Thammarat province located in southern Thailand (8°43′10″N latitude and 99°45′6″E longitude) that has a total area of 720.2 km^2^ and had a population of 33,533 in 2019. The climate in southern Thailand is considered to be tropical. In 2019, the Climatological Center, Thai Meteorological Department, reported that the average temperature in southern Thailand was 27.7°C, with a minimum of 26.4°C in January and a maximum of 28.4°C in April. The annual rainfall was 1990 mm. This district is divided into four subdistricts (tambons), including Nopphitam, Krung Ching, Karo, and Na Reng. Twelve public schools were located in Nopphitam district at the time of the study. The primary occupation is rubber and fruit plantation work, and the majority of the population is Buddhist.

### 2.2. Sample Collection

A total of 120 soil samples were collected from public schools in Nopphitam district between August and September 2017. At each school, ten soil samples were collected comprised of two samples from five different locations where children preferably played games or engaged in activities, including (1) playgrounds, (2) football fields, (3) sidewalks, (4) schoolyards, and (5) areas around cafeterias. The two samples from each location were randomly collected from two sites that were at least 20 meters apart, with 100 g of soil collected from a depth of 0–15 cm [26]. All samples were transferred into individually identified sterile plastic bags, sent to the Parasitological Laboratory of Walailak University, and stored at 4°C until analysis.

### 2.3. Detection of *Toxocara* Eggs

The soil samples were examined for *Toxocara* eggs with a modified flotation method using a sucrose solution [26, 30, 31]. In brief, the soil samples were dried overnight at room temperature and filtered through a 150 *μ*m mesh sieve. Approximately 2 g of powdered soil was transferred to a 15 mL conical tube (Corning, USA), suspended in approximately 8 mL of 0.05% Tween-80 solution, and centrifuged at 2000 rpm for 10 minutes. After discarding the supernatant, the test tube containing the sediment was filled to approximately 1 cm from the top with sucrose solution (specific gravity of 1.200), vortexed, and centrifuged again. The test tube was then filled to the very top with sucrose solution and centrifuged at 1800 rpm for 5 minutes. A cover glass was added to the top of each tube and was examined for *Toxocara* eggs under a light microscope at a magnification of 400 × by an experienced medical technologist.

### 2.4. Statistical Analysis

All statistical analyses were carried out using IBM SPSS Statistics for Windows, version 23.0. The rate of *Toxocara* contamination was assessed by determining the percentage with a 95% confidence interval (95% CI). Fisher's exact test was used to compare the rates of *Toxocara* contamination among the subdistricts and sites of specimen collection. A value of *p* < 0.05 was considered significant.

## 3. Results

**Figure 1:**
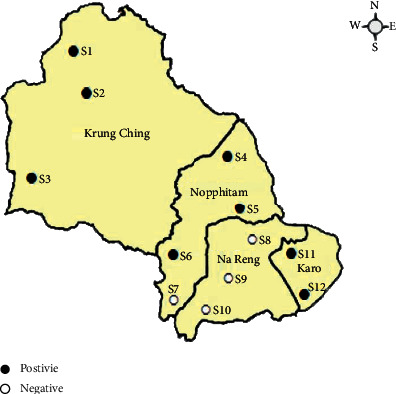
Map of Nopphitam district in Nakhon Si Thammarat province highlighting the distribution of positive (black circle) and negative (white circle) sites for *Toxocara* eggs in soil samples.

This study focused on *Toxocara* egg contamination in the soil of public schools in Nopphitam district using a modified flotation method with a sucrose solution. Our study revealed that *Toxocara* eggs were detected in 8 (66.7%) of the 12 studied public schools in the rural area of Nakhon Si Thammarat province (Figure 1). The contamination rate in each of the subdistricts and public schools is shown in Table 1. Of the 120 soil samples, 22 (18.3%; 95% CI: 11.9, 26.4) soil samples were positive for *Toxocara* eggs. The highest level of soil contamination was observed in Karo subdistrict (6/20, 30%; 95% CI: 11.9, 54.3), followed by Krung Ching subdistrict (8/30, 26.7%; 95% CI: 12.3, 45.9) and Nopphitam subdistrict (8/40, 20.0%, 95% CI: 9.1, 35.6) (Table 1). No *Toxocara* eggs were observed in Na Reng subdistrict. The overall differences in the contamination rates among the subdistricts were significant (*p* < 0.05) (Table 1).

According to the site of specimen collection, playgrounds were observed to have the highest *Toxocara* egg contamination rate (41.7%; 95% CI: 22.1, 63.4), followed by football fields (20.8%; 95% CI: 7.1, 42.2), sidewalks (12.5%; 95% CI: 2.7, 32.4), and schoolyards (12.5%; 95% CI: 2.7, 32.4). The level of soil contamination was the lowest in areas around cafeterias (4.2%; 95% CI: 1.0, 21.1) (Table 2). There were significant differences in the distribution of *Toxocara* eggs between the sites of specimen collection (*p* < 0.05).

## 4. Discussion

According to a previous epidemiological study, soil contamination by *Toxocara* eggs has been observed in many tropical and subtropical regions. To date, data on the contamination rate of *Toxocara* eggs in soil in the southern part of Thailand have been limited. The results of this study showed that 22 out of 120 (18.3%) soil samples from public schools in rural southern Thailand were contaminated with *Toxocara* eggs, which was higher than those observed in previous studies conducted in other countries, such as 7% in various public places in Ardabil city in northwestern Iran [25], 4.75–12.84% in public places in India [19, 20], and 14.9% in urban and rural areas of Poland [32]. The rate of contamination observed in our study was lower than those observed in other parts of the world, including 43% in public schools in the Philippines [17, 33], 28.6% in public parks of Isfahan city in central Iran [21], 45% in public areas of Ahvaz in southwestern Iran [8], 53% in public parks and playground sandpits in Greater Lisbon, Portugal [26], 20.4% in children's play areas in southern England [34], 32–46% in parks in northeastern Poland [23], and 45.8–54.5% in urban and suburban areas of Malaysia [30]. However, the percentage of *Toxocara* egg contamination of soil determined in this study (18.3%) was comparable to that observed in southeastern Asia (21%) and the pooled global rate (21%) reported in a systematic review [14]. In southern Thailand, a previous study conducted in the past decade reported that 19% of samples tested positive for *Toxocara* eggs in Songkhla province [29], a contamination rate that was similar to that observed in our study. Therefore, the reason for the discrepancy in the rate of contamination in previous studies may be due to several factors, including the culture, geographical parameters, climatic conditions, seasonal changes, soil types, type of cat and dog populations, people's attitudes toward pets, sample collection methods, examination methods, and diagnostic techniques [2, 24, 32, 35, 36].

Regarding the sites of specimen collection, the present study showed significant differences in the distribution of *Toxocara* eggs among the sites examined. The highest rate of *Toxocara* egg contamination was observed in playgrounds. Some studies have demonstrated that *Toxocara* eggs have been recovered in sand or soil samples from playgrounds in India [19, 20], Germany [24], and Turkey [37]. In southeastern Asian countries, a previous study conducted in the Philippines demonstrated that 42% of soil samples obtained from a public school were positive for *Toxocara* eggs, and a total of 49% of serum samples from children were positive for *Toxocara* infection [17]. There was a positive correlation between the *Toxocara* egg concentration and the seroprevalence of *Toxocara* infection [17]. A possible explanation for this observation may be that the number of stray dogs and cats has been increasing in rural areas, and they freely roam around school areas and defecate in playgrounds. Therefore, our study results suggest that the playgrounds may be the primary source of transmission of *Toxocara* eggs to humans, especially children. We suggest that schools should limit the access of dogs and cats to school areas, especially playgrounds. Schools should also teach children to avoid playing with soil and discourage geophagia to reduce the rate of *Toxocara* infection. These guidelines are essential for the development of effective programs aimed at minimizing the transmission of toxocariasis to children.

The present study was subjected to the following limitations. We did not collect data regarding other potential factors associated with the contamination rate, such as the details of the soil characteristics, climatic conditions, and seasonal changes. It has been reported that *Toxocara* eggs were most prevalent in low-acidity, relatively high-temperature, and high-moisture soil conditions [2, 3, 33]. Since we did not use molecular techniques in this study, we could not discriminate between *Toxocara* species since the eggs of both *Toxocara* species are similar in size and morphology. Molecular techniques are required for identifying the species of *Toxocara* eggs recovered in soil. Several previous reports have suggested that molecular methods are sufficiently sensitive to detect low levels of parasites and identify different *Toxocara* spp. [8, 16, 38, 39]. Since an assessment of the infection ability of *Toxocara* spp. eggs was not performed in this study, we could not calculate the overall viability rate of the eggs present in soil. This study reflects only the *Toxocara* egg contamination rates in public schools in rural areas; the contamination rates were not compared with those in urban areas. To determine the distribution of *Toxocara* eggs in different environments, further studies should investigate the presence of *Toxocara* eggs in soil samples collected from urban areas or different sampling areas, including public parks, beaches, and roadsides that dogs and cats can freely access.

## 5. Conclusions

This investigation provides baseline knowledge regarding soil contamination with *Toxocara* eggs and important information regarding toxocariasis in southern Thailand. Playgrounds were the most heavily contaminated locations, indicating that playgrounds are a primary source of transmission of *Toxocara* eggs to children. Knowledge of areas contaminated with *Toxocara* eggs is essential for planning effective measures to prevent infection. Teaching children proper handwashing steps and not to eat soil should be implemented to reduce the distribution of *Toxocara* and prevent future *Toxocara* infections.

## Figures and Tables

**Table 1:** Soil contamination by *Toxocara* eggs in the subdistricts and public schools in Nakhon Si Thammarat province in southern Thailand.

Study site	No. of soil samples	No. of positive samples	Rate of contamination (%)	95% CI	Fisher's exact test
Krung Ching					*p*=0.005
S1	10	2	20.0		
S2	10	4	40.0		
S3	10	2	20.0		
Total	30	8	26.7	12.3, 45.9	
Nopphitam					
S4	10	2	20.0		
S5	10	4	40.0		
S6	10	2	20.0		
S7	10	0	0.0		
Total	40	8	20.0	9.1, 35.6	
Na Reng					
S8	10	0	0.0		
S9	10	0	0.0		
S10	10	0	0.0		
Total	10	0	0.0	—	
Karo					
S11	10	4	40.0		
S12	10	2	20.0		
Total	20	6	30.0	11.9, 54.3	

Overall	120	22	18.3	11.9, 26.4	

**Table 2:** Contamination rate of *Toxocara* eggs in the different sampling areas.

Site of collection	No. of soil samples	No. of positive samples	Rate of contamination (%)	95% CI	Fisher's exact test
Playgrounds	24	10	41.7	22.1, 63.4	*p*=0.016
Football fields	24	5	20.8	7.1, 42.2	
Sidewalks	24	3	12.5	2.7, 32.4	
Schoolyards	24	3	12.5	2.7, 32.4	
Areas around cafeterias	24	1	4.2	1.0, 21.1	

Total	120	22	18.3	11.9, 26.4	

## Data Availability

The data used to support the findings of this study are included in the article.
